# Biomedical Imaging Modality Classification Using Combined Visual Features and Textual Terms

**DOI:** 10.1155/2011/241396

**Published:** 2011-09-08

**Authors:** Xian-Hua Han, Yen-Wei Chen

**Affiliations:** ^1^College of Information Science and Engineering, Ritsumeikan University, Kusatsu-Shi, 525-8577, Japan; ^2^College of Information Sciences and Technology, The Pennsylvania State University, University Park, PA 16802, USA

## Abstract

We describe an approach for the automatic modality classification in medical image retrieval task of the 2010 CLEF cross-language image retrieval campaign (ImageCLEF). This paper is focused on the process of feature extraction from medical images and fuses the different extracted visual features and textual feature for modality classification. To extract visual features from the images, we used histogram descriptor of edge, gray, or color intensity and block-based variation as global features and SIFT histogram as local feature. For textual feature of image representation, the binary histogram of some predefined vocabulary words from image captions is used. Then, we combine the different features using normalized kernel functions for SVM classification. Furthermore, for some easy misclassified modality pairs such as CT and MR or PET and NM modalities, a local classifier is used for distinguishing samples in the pair modality to improve performance. The proposed strategy is evaluated with the provided modality dataset by ImageCLEF 2010.

## 1. Introduction

Imaging modality is an important aspect of the image for medical retrieval [1–6]. “In user studies, clinicians have indicated that modality is one of the most important filters that they would like to be able to limit their search by. Many image retrieval websites (Goldminer, Yottalook) allow users to limit the search results to a particular modality. However, this modality is typically extracted from the caption and is often not correct or present” [7]. Some works have shown that image modality can be extracted from the image itself using visual features [8–10]. Therefore, in this paper, we propose to use both visual and textual features for medical image representation, and combine the different features using normalized kernel function in SVM.

In computer vision, studies have shown that the simple global features such as histogram of edge, gray or color intensity, can represent images, and give the acceptable performance in image retrieval or recognition research fields. Based on the success of the above-mentioned visual features for general image recognition, we also use them as medical image representation for modality classification. Recently, using local visual feature for image representation has become very popular, and been proved to be very effective for image categorization or retrieval [11]. The most famous approach for image representation using local visual feature is bag of keypoints [12, 13]. The basic idea of bag of keypoints is that a set of local image patches is sampled using some method (e.g., densely, randomly, or using a keypoint detector) and a vector of visual descriptors is evaluated on each patch independently (e.g., SIFT descriptor, normalized pixel values). The resulting distribution of descriptors in descriptor space is then quantified in some way (e.g., by using vector quantization against a prespecified codebook to convert it to a histogram of votes for (i.e., patches assigned to codebook centres) and the resulting global descriptor vector is used as a characterization of the image (e.g., as feature vector on which to learn an image classification rule based on an SVM classifier). Furthermore, according to the visual properties of medical images, we also calculate a histogram of small-block variance as visual feature for image representation. For textual feature, we predefine 90 vocabulary words somewhat according to the statistical properties of training samples' captions and our knowledge about medical modality, and calculate a binary histogram for any medical image using their captions. After obtaining the different features for image representation, we combine them together using kernel function for SVM classifier. Because different features maybe have deferent scale and dimension, in order to allow each individual feature to contribute equally for modality classification, we normalize the distance between two samples using mean distance of all training samples, and then, obtain the kernel function for each individual feature. The final kernel for SVM classification is the mean of individual kernel, which can be called Joint Kernel Equal Contribution (JKEC). Furthermore, for some easy misclassified modalities such as CT and MR or PET and NM, a global classifier, which deals with all modalities in the used database, may not be effective in distinguishing the local modalities from each other. Therefore, after the global classification, a local classifier is used in the easy misclassified modality pairs to refine the classification results. Finally, the proposed algorithm is evaluated on the modality dataset of ImageCLEF 2010, and almost achieve the expected accuracy rate expected by the modality classification task of ImageCLEF 2010, which is about 97% classification rate.

## 2. Feature Extraction for Image Representation

In this section we describe how we extract a feature representation, which is somewhat robust to the high variability inherent in medical images and includes enough discriminative information for modality category. Some previous studies showed that it is difficult to correctly classify image categorization with only one type of image feature [14, 15]. So in this paper, we represent images with different images features including gray and color intensity histogram, block-based edge and variance histogram, popular bag-of-words model as visual feature, and a binary histogram of the predefined vocabulary words from image captions as textual feature. Then we merge them together for modality classification. Next, we simply introduce the used features for medical image representation.

### 2.1. Visual Features

#### 2.1.1. Gray and Color Intensity Histogram

Intensity histograms are widely used to capture the distribution information in an image. They are easy to compute and tend to be robust against small changes of camera viewpoints. For Gray intensity histogram, we can calculate the number of each intensity (0–255) for all image pixel, and normalize it using pixel number. Given an image **I** in some color space (e.g., red, green, and blue), to calculate color histogram the color channels are quantized into a coarser space with *k* bins for red, *m* bins for green, and *l* bins for blue. Therefore the color histogram is a vector **h** = (*h*_1_,*h*_2_,…,*h*_*n*_)^*T*^, where *n* = *kml*, and each element *h*_*i*_ represents the number of pixels of the discretized color in the image. We assume that all images have been scaled to the same size. Otherwise, we normalize histogram elements as

(1) $$h_{j}^{\prime }=\frac{y_{j}}{\sum_{j=0}^{n}y_{j}}.$$

#### 2.1.2. Block-Based Edge Histogram

We firstly segment the image into several blocks, and calculate edge histogram weighted by gradient intensity in each block [16]. In experiment, we grid-segment an image into 4-by-4 block, and calculate a 20-bin edge histogram in each block. So we have 320-(20∗16-)dimensional edge histogram feature for medical image representation.

#### 2.1.3. Block-Based Variance Histogram

For each pixel in an image, a small patch centered by the specific pixel are used for calculating the local variation of the pixel. After obtaining the local variation of all pixels in the image, a histogram of variation intensity is calculated for the image representation.

#### 2.1.4. Bag-of-Words Feature

**Figure 1:**
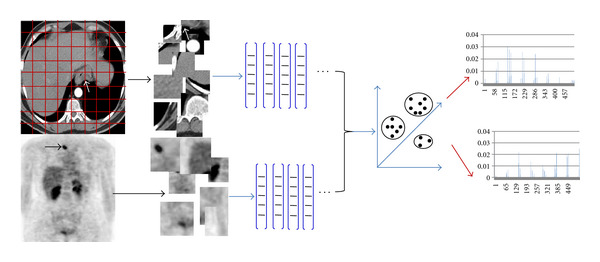
BOW feature extraction procedure.

In computer vision, local descriptors (i.e., features computed over limited spatial support) have proved well-adapted to matching and recognition tasks, as they are robust to partial visibility and clutter. In this paper, we use grid-sampling patches, and then compute appearance-based descriptors on the patches. In contrast to the interest points from the detector, these points can also fall onto very homogeneous areas of the image. After the patches are extracted, the SIFT [11] descriptor is applied to represent the local features. The SIFT descriptor computes a gradient orientation histogram within the support region. For each of 8 orientation planes, the gradient image is sampled over a 4-by-4 grid of locations, thus resulting in a 128-dimensional feature vector for each region. A Gaussian window function is used to assign a weight to the magnitude of each sample point. This makes the descriptor less sensitive to small changes in the position of the support region and puts more emphasis on the gradients that are near the center of the region. To obtain robustness to illumination changes, the descriptors are made invariant to illumination transformations of the form *aI*(*x*) + *b* by scaling the norm of each descriptor to unity [11]. These SIFT features are then clustered with a *k*-means algorithm using the Euclidean distance. Then we discard all information for each patch except its corresponding closest cluster center identifier. For the test data, this identifier is determined by evaluating the Euclidean distance to all cluster centers for each patch. Thus, the clustering assigns a cluster *c*(*x*)(*c* = 1,…*C*) to each image patch *x* and allows us to create histograms of cluster frequencies by counting how many of the extracted patches belong to each of the clusters. The histogram representation *h*(*X*) with *C* bins is then determined by counting and normalization such that

(2) $$h_{c}\left(X\right)=\frac{1}{L_{X}}\overset{L_{X}}{\underset{l=1}{\sum }}\delta \left(c,c\left(x_{l}\right)\right),$$

where *δ* denotes the Kronecker delta function.  Figure 1 shows the procedure of bag-of-words (BoW) feature extraction and the extracted histogram feature of an example image. Obviously, there exist alternatives to algorithmic choices made in the proposed method. For example, different interest point detectors can be used. However, it does not manifest obvious merit for different background cluster of images. Furthermore, the geometrical relation between the extracted patches is completely neglected in the approach presented here. While this relation could be used to improve classification accuracy, it remains difficult to achieve an effective reduction of the error rate in various situations by doing so.

### 2.2. Textual Features

**Figure 2:**
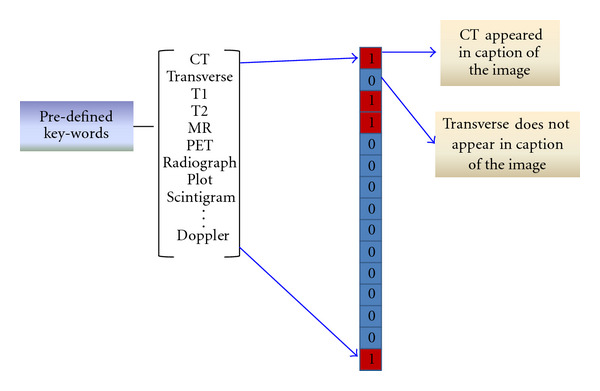
Textual feature extraction procedure.

According to the statistical properties of word occurrence in each training modality image's captions and our prior knowledge about the classifying modalities, we select 90 key-words, such as CT, curve, MR, urethrogram, and PET, as the vocabulary for forming a binary histogram for each medical image. The binary histogram for image representation is 90-dimension vector, where each dimension is correspond to one selected keyword. If one keyword appeared one or more than one time in an image's caption, the value of the corresponding dimension in its represented binary histogram will be 1, otherwise it will be 0. The textual feature extraction procedure is illustrated in Figure 2.

## 3. Feature Fusion

Given a training set (*x*_*i*_,*y*_*i*_)_*i*=1,2,…,*N*_ of *N* instances consisting of an image *x*_*i*_ ∈ *χ* and a class label *y*_*i*_ ∈ 1,2,…, *C*, and given a set of *F* image features *f*_*m*_ : *χ* → *ℜ*^*d*_*m*_^, *m* = 1,2,…, *F*, where *d*_*m*_ denotes the dimensionality of the *m*th feature, the problem of learning a classification function *y* : *χ* → 1,2,…, *C* from the features and training set is called feature combination problem. In computer vision, the problem of learning a multiclass classifier from training data is often addressed by means of kernel methods. Kernel methods make use of kernel functions defining a measure of similarity between pairs of instances. In the context of feature combination it is useful to associate a kernel to each image feature as the following equation (3), and combine the kernels of different features together. For a kernel function *K* of each feature between real vectors we define the short-hand notation:

(3) $$K_{m}\left(I_{i},I_{j},\right)=K\left(f_{m}\left(I_{i}\right),f_{m}\left(I_{j}\right)\right)=K\left(S\left(f_{m}\left(I_{i}\right),f_{m}\left(I_{j}\right)\right)\right),$$

where **I**_*i*_ and **I**_*j*_ are two samples, *f*_*m*_(**I**_*i*_) is the *m*th extracted feature from the sample **I**_*i*_, and *S*(*f*_*m*_(**I**_*i*_), *f*_*m*_(**I**_*j*_)) is the similarity measure between the *m*th features of the samples **I**_*i*_ and **I**_*j*_. Then the image kernel *K*_*m*_: *χ* × *χ* ∈ *ℜ* only considers similarity with respect to image feature *f*_*m*_. If the image feature is specific to a certain aspect, say, it only considers color information, then the kernel measures similarity only with regard to this aspect. The subscript *m* of the kernel can then be understood as indexing into the set of features. Because different features maybe have different scale and dimension, in order to allow each individual feature to contribute equally for modality classification, we normalize the distance between two samples using mean distance of all training samples, and then, obtain the kernel function for each individual feature *f*_*m*_. The final kernel for SVM classification is the mean of individual kernel, which can be called Joint Kernel Equal Contribution (JKEC). For the feature similarity calculation of two samples, we use several distances: Euclidean distance (*L*_2_ distance), *L*_1_ distance, and *χ*^2^ distance, for evaluating the classification performance. The *χ*^2^ distance for two samples can be calculated as follows:

(4) $$S_{m}^{i,j}=S\left(f_{m}\left(I_{i}\right),f_{m}\left(I_{j}\right)\right)=\overset{L}{\underset{1}{\sum }}\frac{{\left(x_{l}-y_{l}\right)}^{2}}{x_{l}+y_{l}},$$

where *x* and *y* represent the *m*th features *f*_*m*_(**I**_*i*_), *f*_*m*_(**I**_*j*_) of samples *i* and *j*, respectively, and *x*_*l*_ is the *l*th element of the vector *x*. *S*_*m*_^*i*,*j*^ is the similarity measure of the *m*th feature between the *i*th and *j*th training samples. Then, the RBF function is used for calculating the kernel:

(5) $$K_{m}^{i,j}=K_{m}\left(I_{i},I_{j},\right)=\exp\;\;\left(\frac{-S\left(f_{m}\left(I_{i}\right),f_{m}\left(I_{j}\right)\right)}{\gamma_{m}}\right),$$

where *γ*_*m*_ is the normalized item for kernel function of the *m*th feature. Here, we use the distance mean of all training samples as *γ*_*m*_ = 1/*N*^2^(∑_*i*_^*N*^∑_*i*_^*N*^*S*_*m*_^*i*,*j*^) (*N* is the training sample number), which will lead to similar contribution of each feature to kernel. Then the final combined kernel function can be obtained by

(6) $$K^{i,j}=\frac{1}{M}\overset{M}{\underset{i}{\sum }}K_{m}^{i,j},$$

where *M* is the feature number for image representation. The proposed algorithm is evaluated on the modality training dataset of ImageCLEF 2010, which expects about 97% classification rate on the released evaluated and test datasets. Because the ground-truths of the evaluated and test dataset are not released, we cross-validate our proposed strategy with the released training dataset firstly. The classification rate with our experiment on training dataset almost approximated the required goal of the modality classification task.

## 4. Refinement Procedure for Easy Misclassified Modalities

In the released medical database by ImageCLEF 2011, some modalities have a lot of visual similarity such as PET and NM modality. Therefore, it is difficult to distinguish them in the global modality classification, which deals with all modalities in the database. In this section, after the global conventional classification, we design local classifiers to refine the classification results in easy-misclassified modalities. Next, we firstly explain the used dataset, and then, introduce how to design the local classifier according to evaluation results.

### 4.1. Image Data

**Figure 3:**
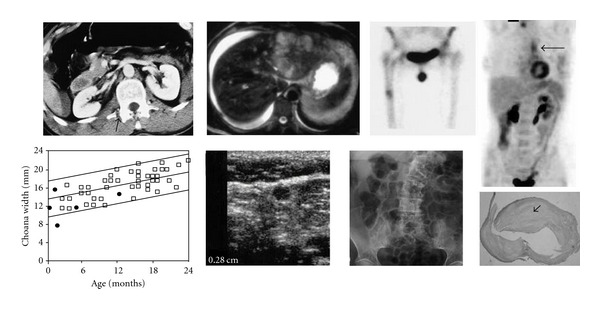
Sample images of 8 medical modalities. From left to right and top to bottom, the images are CT, MR, NM, PET, GX, US, XR, and PX, respectively.

The database released for the ImageCLEF-2010 Medical modality classification in medical retrieval task includes 2390 annotated modality images (CT: 314; GX: 355; MR: 299; NM: 204; PET: 285; PX: 330; US: 307; XR: 296) for training and a separate evaluated set consisting of 2620 images. The aim is to automatically classify the evaluated set using 8 different modality label sets including CT, MR, and PET. Some example images are shown in Figure 3. A more detailed explanation of the database and the tasks can be found in [17].

### 4.2. Local Classifier Designing

**Figure 4:**
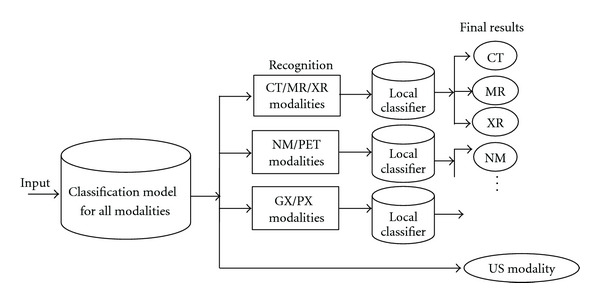
The refinement procedure with the local classifiers.

**Figure 5:**
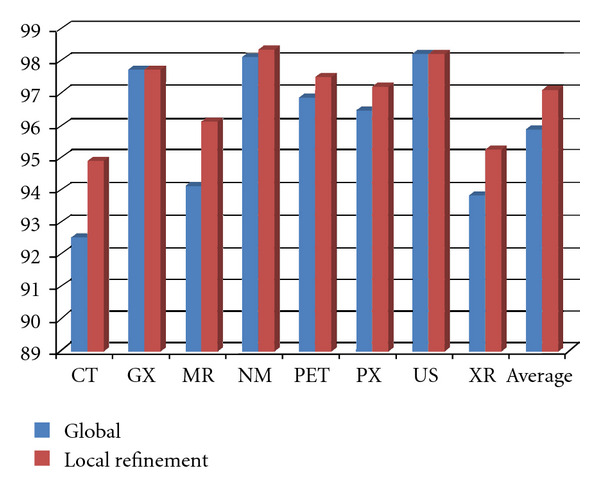
The compared recognition rates for all modalities. Blue bar: without refinement using local classifiers; Red bar: with refinement.

For validating the discriminant properties of different modalities, we randomly select 180 samples from each medical modality in ImageCLEF 2010 training dataset, and the remainder for testing. We combine all visual and textual features using the JKEC fusion strategy introduced in Section 3, for modality classification. The confusion matrix of one run is shown in Table 1. From Table 1, it can be seen that 92.537% CT sample images are correctly recognized as CT modality, and 3.9851% and 2.2388% are classified as MR and XR modalities, respectively. On the other hand, about 2-3% MR or XR sample images are also misrecognized as CT modality. At the same time, it is obvious that NM and PET or GX and PX modalities are also easily misclassified from each other. Therefore, we design three local classifiers for the limited easy-misclassified modalities, which are CT, MR, and XR group, NM and PET group, GX and PX group, to refine the classification results in local regions. The refinement procedure with the local classifiers are shown in Figure 4. The compared experimental results with or without refinement procedure are shown in Figure 5. From Figure 5, it can be seen that the recognition rates for CT, MR, PET, PX, and XR modalities with local classifier refinement can be improved more than 1% compared to those without refinement.

## 5. Experiments

In this section, we validate the recognition rates of different features with three types of similarity measures: Euclidean distance (*L*_2_ distance), *L*_1_ distance and *χ*^2^ distance, and do the cross-validation experiments using the combined visual and textual features on ImageCLEF 2010 training dataset. Then, the submitted runs to medical modality classification of ImageCLEF 2010 and the released results will be introduced.

**Figure 6:**
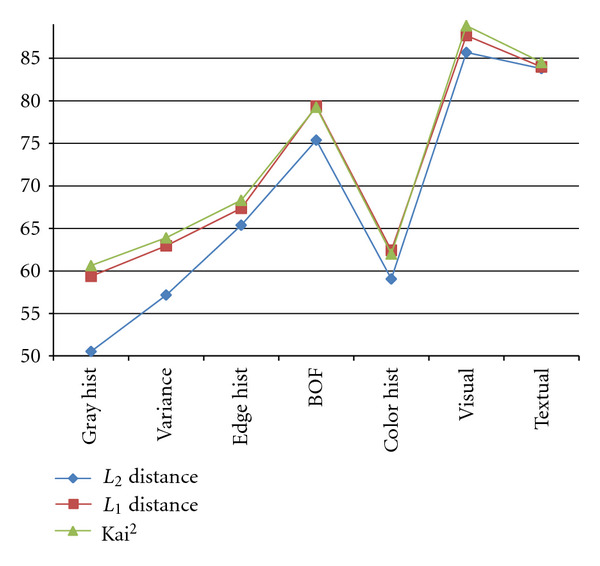
The compared recognition rates with different features using three types of similarity measures: Euclidean distance (*L*_2_ distance), *L*_1_ distance, and *χ*^2^ distance. Gray hist, variance, edge hist and color hist represent the histogram of gray, variance, edge, and color, respectively; BOF and textual mean the BOF and textual features.

**Figure 7:**
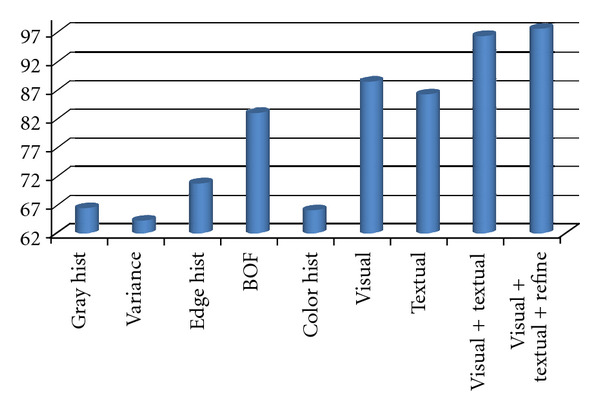
The recognition rates of cross-validation strategy using different features, where visual means the recognition rate using the combined feature of all visual ones, visual + textual means those using the combined textual and visual features, and visual + textual + refine means using refinement procedure after classification with the combined textual and visual features.

1. The recognition rates of different features with three types of similarity measures: in order to validate what kind of distance measure is adaptive to each extracted feature for image representation, we apply three types of similarity measures: Euclidean distance (*L*_2_ distance), *L*_1_ distance, and *χ*^2^ distance, for calculating the kernel function as in (5) of SVM classifier. In the experiments, with the ImageCLEF 2010 training dataset, 180 images are randomly selected for training from each modality, the remainder are for test. The compared recognition rates are shown in Figure 6, where “Kai2” means *χ*^2^ distance. From Figure 6, it can be seen that *L*_1_ and *χ*^2^ distance can obtain much better performance than *L*_2_ distance for most features, and *χ*^2^ distance can achieve a little better than or similar results to *L*_1_ distance. Then, in the next experiments, we utilize *χ*^2^ distance for a similarity measure of all features to calculate SVM kernel functions.
2. Cross-validation experiments: in the experiments, we firstly divide the training dataset of ImageCLEF 2010 into 5 groups, and use 4 groups as training and 1 group as test to cross-validate the performance of different features with *χ*^2^ distance as similarity measure. The recognition rates are shown, Figure 7, where visual means with the combined features of all visual ones, Visual + Textual means with the combined features of all visual and textual ones, visual + textual + refine means using refinement procedure after classification with all combined features. From Figure 7, it can be seen that the average recognition rate can be improved about 1% after the refinement procedure introduced in Section 4.
3. Submitted runs: as Medical Image Processing Group (MIPG) of our Intelligent Image Processing Laboratory (IIPL) in Ritsumeikan University, we prepared four runs for evaluation image set, which used combine visual feature, textual feature, both visual and textual features, and weighted visual and textual features. The recognition results are shown in Table 2. We submitted two runs using textual, combined textual and visual features by on-line-system, respectively. Our results are ranked second among 6 participating teams, and the result of one run is also ranked second among about 50 runs [18]. At the same time, the recognition rates (submitted run: 93.36%, unsubmitted run: 93.89%) of our methods using mixed feature (Visual plus textual) are similar to the first ranking results 94% by Xerox Research Centre Europe.

## 6. Conclusions

In this paper, we proposed to extract different visual and textual features for medical image representation, and use JKEC strategy to fusion them for modality classification. To extract visual features from the images, we used histogram descriptor of edge, gray, or color intensity and block-based variation as global features and SIFT histogram as local feature, and the binary histogram of some predefined vocabulary words for image captions is used for textual feature. Because different features maybe have different scale and dimension, in order to allow each individual feature to contribute equally for modality classification, we proposed to use joint kernel equal contribution (JKEC) for kernel fusion of different features. Furthermore, for some easy misclassified modality pairs such as CT and MR or PET and NM modalities, a local classifier is used for distinguishing samples in the pair modality to improve performance. The proposed algorithm is evaluated by the provided modality dataset by ImageCLEF 2010.

## Figures and Tables

**Table 1:** Confusion matrix of one run on medical evaluated dataset using combined visual and texture features.

Modality	CT	GX	MR	NM	PET	PX	US	XR
CT (%)	92.537	0	3.9851	0	0.4925	0.7463	0	2.2388
GX (%)	0	97.714	0	0	0	0.2857	0	0
MR (%)	3.3613	0	94.118	0	0	0.8403	0	1.6807
NM (%)	0	0	0	98.23	1.77	0	0	0
PET (%)	0	0	0.4224	2.53	97.048	0	0	0
PX (%)	0	1.333	0	0.6667	0	96.667	0	1.3333
PX (%)	0	0	0.7874	0	0.7874	98.425	0	0
XR (%)	1.7241	0	2.5862	0.8621	0	0.8621	0	93.966

**Table 2:** Overall classification rates on medical evaluated dataset using combination of different features.

Features	Visual	Textual	Visual + texture	Weighted visual + textual
Classification rate (%)	87.07	84.58	93.36	93.89
